# Case Report: A Challenging Superior Hypogastric Plexus Block in a Patient with Enlarged and Bifid Transverse Processes

**DOI:** 10.5812/aapm-163195

**Published:** 2025-07-26

**Authors:** Thuraya Al Hadifi, Ali Al Abadi

**Affiliations:** 1 Department of Anesthesia and ICU and Pain Medicine, Sultan Qaboos University Hospital, Muscat, Oman

**Keywords:** Hypogastric Plexus, Chronic Pelvic Pain, Endometriosis, Female

## Abstract

**Introduction:**

Superior hypogastric plexus block (SHPB) is an established treatment for chronic pelvic pain (CPP). Anatomical variations can significantly complicate interventional pain procedures.

**Case Presentation:**

We present a case of a 44-year-old woman with CPP secondary to endometriosis and unique lumbosacral (L-S) anatomy, including enlarged and bifid transverse processes, which posed a challenge to standard SHPB techniques. This necessitated a tailored approach to ensure success and patient safety. Successful bilateral blockade was achieved using a combination of posterolateral and trans-discal approaches under fluoroscopic guidance. The patient reported substantial pain relief and improved quality of life.

**Conclusions:**

This case underscores the clinical relevance of recognizing and adapting to anatomical variations during SHPB to optimize procedural success and patient outcomes. Despite the limitations inherent in its retrospective design and reliance on existing clinical data, this study reinforces the need for individualized approaches in similar interventions.

## 1. Introduction

Endometriosis is a chronic inflammatory condition affecting women of reproductive age, characterized by the presence of endometrial tissue outside the uterine cavity (1). Its pathophysiology involves complex interactions of inflammation, hormonal influences, and nerve sensitization, often leading to severe and debilitating pain. A significant manifestation of this and other conditions is chronic pelvic pain (CPP) (2, 3). This type of pain is typically described as cyclic or non-cyclic pelvic pain that is persistent, disabling, or intermittently experienced within the pelvis, enduring for at least six months, and may be accompanied by symptoms like painful menstruation (dysmenorrhea), painful intercourse (dyspareunia), painful urination (dysuria), or painful defecation (dyschezia).

Globally, CPP affects up to 25% of females of reproductive age and 15% of all females. Its etiology is diverse, often classified by the affected organ system, and it frequently coexists with conditions such as irritable bowel syndrome, major depressive disorder, anxiety, fibromyalgia, and interstitial cystitis (4). The persistent burden of CPP severely impacts patients’ quality of life, manifesting as considerable functional impairment, psychological distress, and limitations across their daily activities, professional lives, and social interactions.

The management of CPP typically follows a multimodal, multidisciplinary, stepped-care approach. Initial strategies often involve conservative medical management (including hormonal therapies and analgesics), lifestyle modifications, specialized physical therapy, and psychological support. When these measures provide insufficient relief, the management plan may escalate to include surgical intervention (depending on etiology) or interventional pain management techniques, such as the superior hypogastric plexus block (SHPB) and ganglion of impar block (GOIB), may be considered.

The SHPB, which targets a sympathetic nerve plexus involved in pelvic pain transmissions, is a well-established interventional pain management technique for treating CPP (5, 6). Although generally considered safe and effective, anatomical variations can pose challenges during the procedure, potentially increasing the risk of complications and decreasing the likelihood of successful block placement. This case report highlights the management of a patient with enlarged and bifid transverse processes, which presented a unique challenge during SHPB.

## 2. Case Presentation

A 44-year-old nulliparous divorced woman presented with a history of stage 4 endometriosis and debilitating CPP refractory to hormonal therapy. She described her pain as constant, deep, and cramping in the lower abdomen and pelvis, often radiating to her lower back and thighs. Additionally, she reported significant dyspareunia, dyschezia, and occasional dysuria. These symptoms profoundly interfered with her sleep, work, social interactions, and intimate relationships.

She underwent multiple surgeries related to endometriosis. Her pain management regimen included tramadol, pregabalin, and amitriptyline. She was also engaged in pelvic floor physiotherapy and psychosocial support. Despite ongoing pelvic floor physical therapy and cognitive behavioral therapy, her pain remained poorly controlled.

Given the patient’s refractory pain and the failure of previous medical management, interventional pain management techniques were considered. The SHPB and GOIB were evaluated as potential treatment options. The patient was informed about the procedure, its risks, and benefits. After obtaining informed consent, SHPB and GOIB were performed under fluoroscopic guidance.

**Figure 1.**
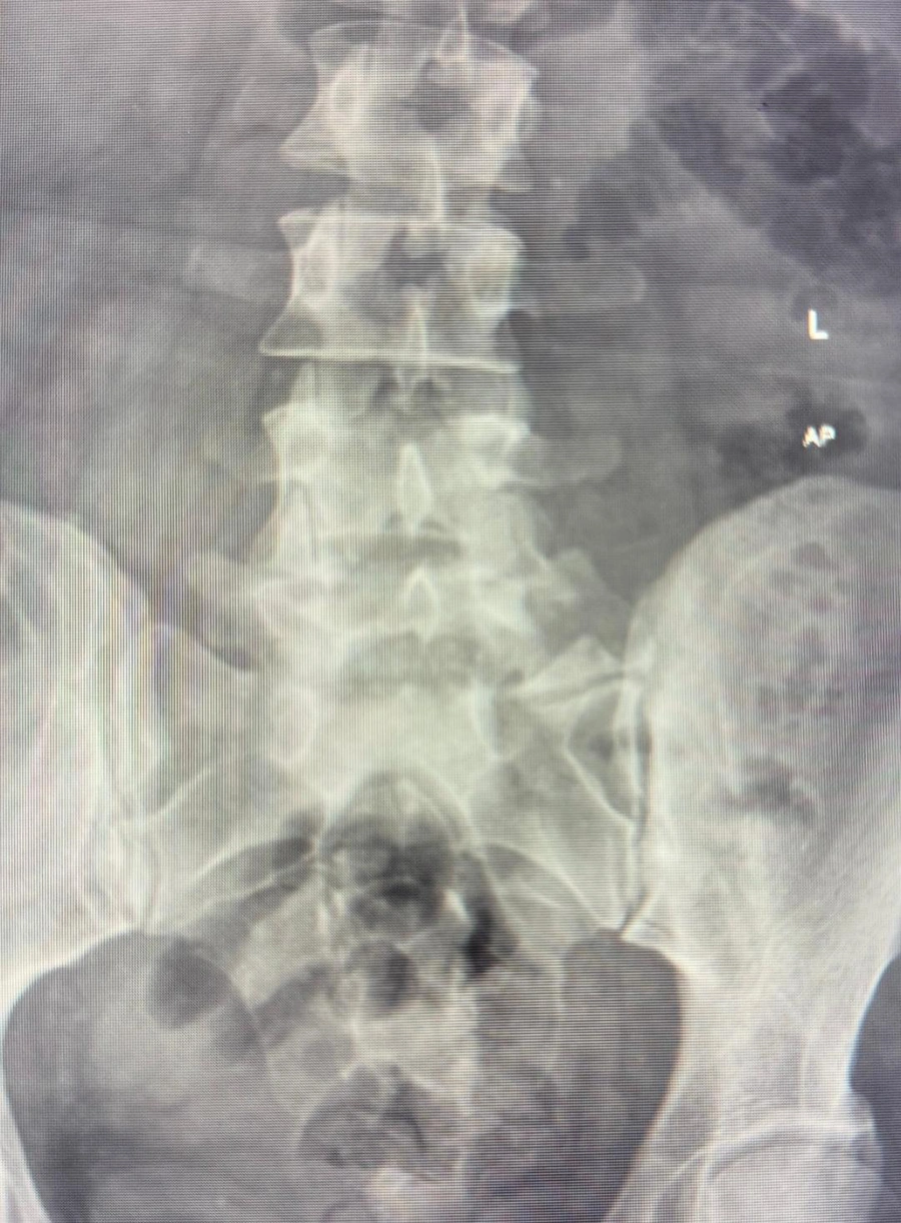
Anteroposterior (AP) view of the lumbosacral (L-S) spine. This image reveals a bifid transverse process on the right side at the L5 vertebral level. In contrast, the left transverse process of L5 appears distinctly enlarged compared to the right side and the transverse processes of the adjacent vertebrae.

A review of the pre-procedure lumbosacral (L-S) X-rays identified a bifid right L5 transverse process and an enlarged left transverse process (Figure 1). Based on these findings, a right-sided posterolateral approach was selected, with the goal of achieving bilateral spread of contrast. A left-sided trans-discal approach was planned as a contingency in case the attempt resulted in unilateral contrast distribution (7, 8).

### 2.1. Procedural Course

Prior to the procedure, 2 g of cefazolin was given intravenously for discitis prophylaxis (9). Under fluoroscopic guidance and sterile conditions in the operating room, the patient was positioned prone with a pillow beneath the iliac crest to decrease lumbar lordosis. Intravenous sedation was provided with increments of midazolam totaling 3 mg and fentanyl totaling 50 mcg. The vital signs were continuously monitored.

**Figure 2.**
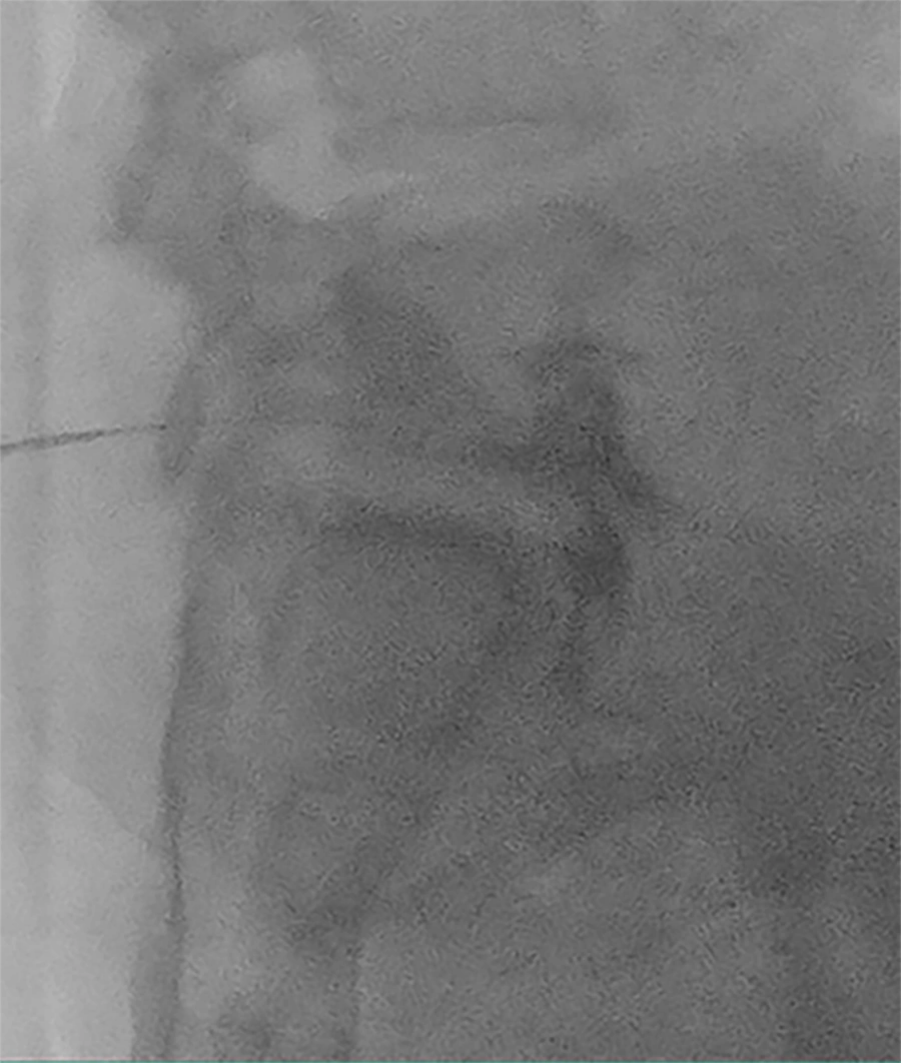
Fluoroscopic view of the right-sided posterolateral approach. This lateral view shows contrast spread in a craniocaudal direction anterior to the L5-S1 disc.

Fluoroscopic imaging was utilized to visualize the L-S spine. Anteroposterior (AP) views were obtained. Subsequently, an oblique C-arm tilt was used to achieve a ‘scotty dog view’ by overlapping the L5 spinous process with the contralateral facet line. Finally, a cephalad C-arm tilt was performed to displace the iliac crest from the field of view. After local anesthetic infiltration of the skin and subcutaneous tissue, a 20-gauge, 150 mm spinal needle with a curved tip was advanced. The needle entry point was established lateral to the lower L5 vertebral body, just cephalad to the iliac crest. A coaxial approach was employed to contact the lower L5 vertebral body. Lateral fluoroscopic imaging confirmed needle depth, and the needle was then wiggled anteriorly until positioned slightly anterior to the L5-S1 disc. The AP fluoroscopy view verified the needle tip location at the junction of the lateral and mid-third of the vertebral body. Contrast injection with Omnipaque 300 demonstrated unilateral (right-sided) spread anterior to the L5-S1 disc (Figure 2). A total of 10 mL of 0.25% bupivacaine mixed with 4 mg of dexamethasone was administered.

For complete coverage of the superior hypogastric plexus, a left-sided procedure was attempted. Due to anatomical variation, specifically an enlarged left transverse process, the intended posterolateral approach was not technically feasible. Consequently, a trans-discal approach was utilized. Fluoroscopic guidance aimed to provide an unobstructed view of the anterolateral L5-S1 disc. This was accomplished by obtaining AP views and squaring off the lower L5 and upper S1 endplates. An oblique C-arm tilt, approximately 20°, was used to clear the S1 superior articulating process (SAP) and lamina from the needle path.

**Figure 3.**
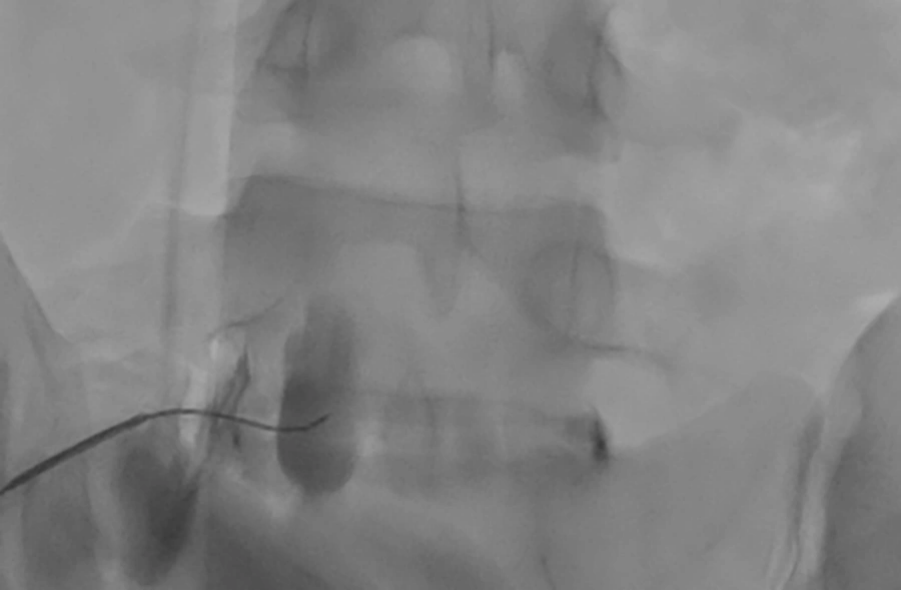
AP fluoroscopic view illustrating the left-sided trans-discal approach and the unilateral anterior spread of contrast

The entry point was positioned just lateral to the S1 SAP. Excessive obliquity was avoided to prevent the iliac crest from obscuring the view and to prevent nucleus pulposus penetration. A double-needle technique, employing an 18G introducer with a 22G needle, was considered to reduce the risk of discitis. A coaxial view was utilized to guide the needle to the posterolateral aspect of the L5-S1 disc. AP and lateral fluoroscopy views confirmed the appropriate needle trajectory. Then, the needle was advanced through the lateral third of the disc, extending just anterior to its border. Loss of resistance confirmed needle passage beyond the disc. Contrast injection demonstrated anterior spread (Figure 3). A total of 10 mL of 0.25% bupivacaine mixed with 4 mg dexamethasone was injected uneventfully.

### 2.2. Post-procedural Course

The patient experienced immediate and substantial pain relief. Her visual analog score (VAS) decreased from 8 to 4 within one hour of the block. During the follow-up appointment, she reported a sustained reduction in lower abdominal pain and a marked improvement in quality of life. The pain relief persisted for approximately two months, at which point the patient’s pain gradually recurred. During her visit, written informed consent was obtained from the patient for the publication of this case report.

## 3. Discussion

The CPP is a debilitating condition that significantly impacts the quality of life of many women. While endometriosis is a common cause, CPP can also arise from various other etiologies (5, 10). The SHPB represents a well-established interventional modality in the management of CPP (11). The superior hypogastric plexus is a retroperitoneal network of nerves located anterior to the L5 vertebral body and S1 vertebrae (12). It primarily contains sympathetic fibers that carry pain signals from pelvic viscera, including the uterus, ovaries, prostate, distal colon, rectum, and bladder (13). Blocking this plexus can interrupt the transmission of pain signals, thus providing significant relief for selected patients.

The SHPBs are conventionally performed in the prone position utilizing either a posterior or trans-discal approach. While modified techniques and alternative patient positioning have been described, these established approaches remain the mainstay, offering improved visualization during needle placement and potentially lowering the risk of complications. Tavakoli et al. described performing the SHPB with patients in the lateral decubitus position, suggesting it as a more tolerable alternative when the prone position is not feasible (14).

Anatomical variations, as demonstrated in our case, can make the traditional posterior approach to SHPB technically challenging. It may cause increased patient discomfort during needle manipulation and often requires a more oblique trajectory to overcome the enlarged transverse process, thereby increasing the risk of injury to surrounding structures. In contrast, the trans-discal approach offers a more direct route to the plexus, potentially simplifying the procedure, reducing procedure time, and improving accuracy, especially in cases with difficult anatomy. However, this approach introduces the risk of disc penetration, raising concerns about potential complications such as discitis or accelerated disc degeneration. While the available literature suggests these complications are infrequent, they remain a consideration (8).

Ultimately, the choice between these approaches necessitates careful consideration of the patient’s specific anatomy, the practitioner’s experience, and a thorough risk-benefit assessment.

The importance of pre-procedural imaging, such as L-S X-ray, computed tomography (CT), or magnetic resonance imaging (MRI), is emphasized in this paper, particularly for cases involving anatomical variations like enlarged and bifid transverse processes. Pre-procedural imaging enables practitioners to comprehensively visualize the patient’s unique spinal anatomy. This allows for the identification of any potential anatomical variations or challenges, thereby allowing the tailoring of the procedure to the individual patient, ultimately improving the safety and efficacy of the intervention.

### 3.1. Conclusions

The SHPB is a valuable therapeutic option for patients with CPP. However, anatomical variations can pose significant challenges to accurately targeting the superior hypogastric plexus. By carefully considering patient anatomy, utilizing appropriate imaging techniques, and adapting the procedural approach, pain physicians can optimize SHPB success and improve patient outcomes. This case reinforces the need for individualized approaches in similar interventions, despite the limitations inherent in single case reports.

## Data Availability

The data presented in this case study are uploaded during submission as a supplementary file and are openly available for readers upon request.
